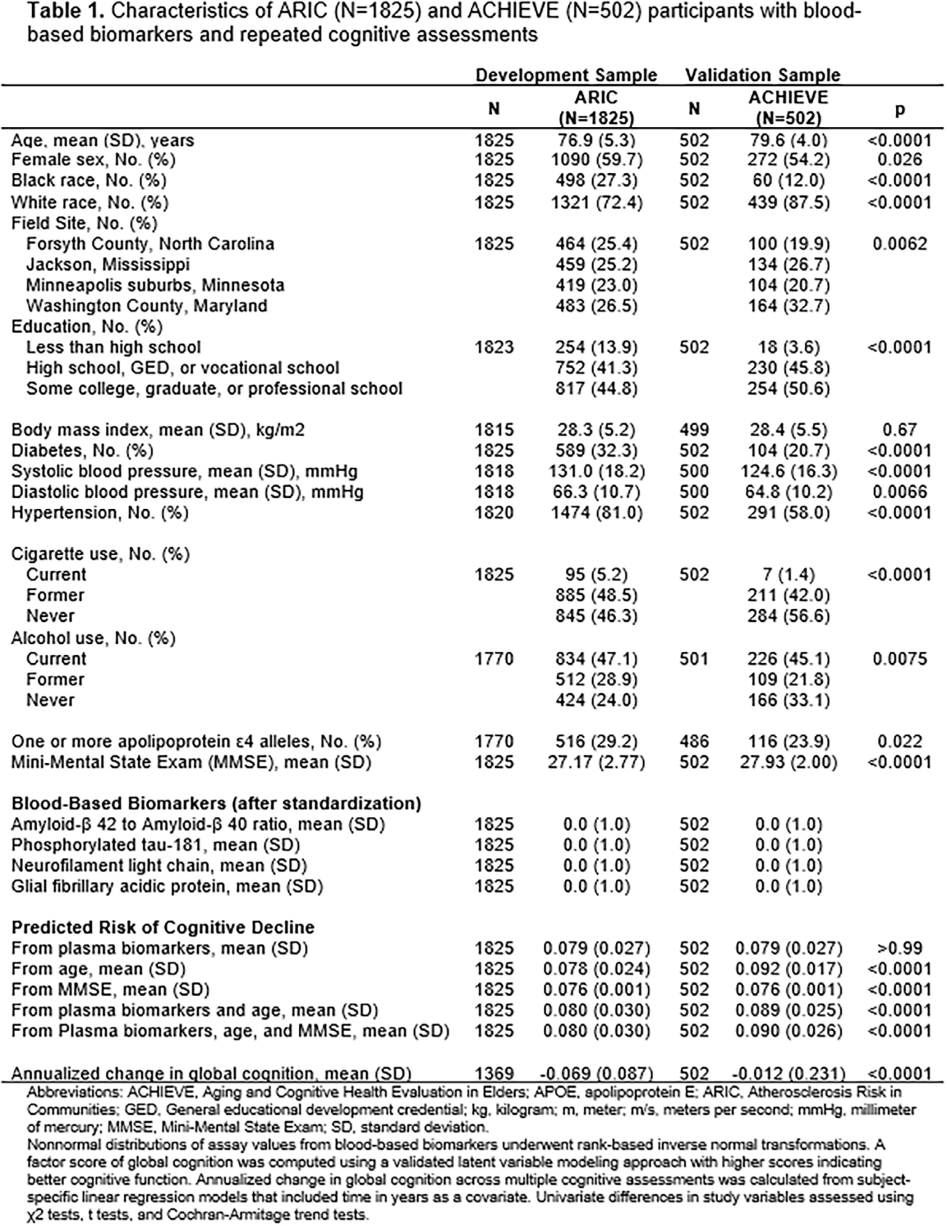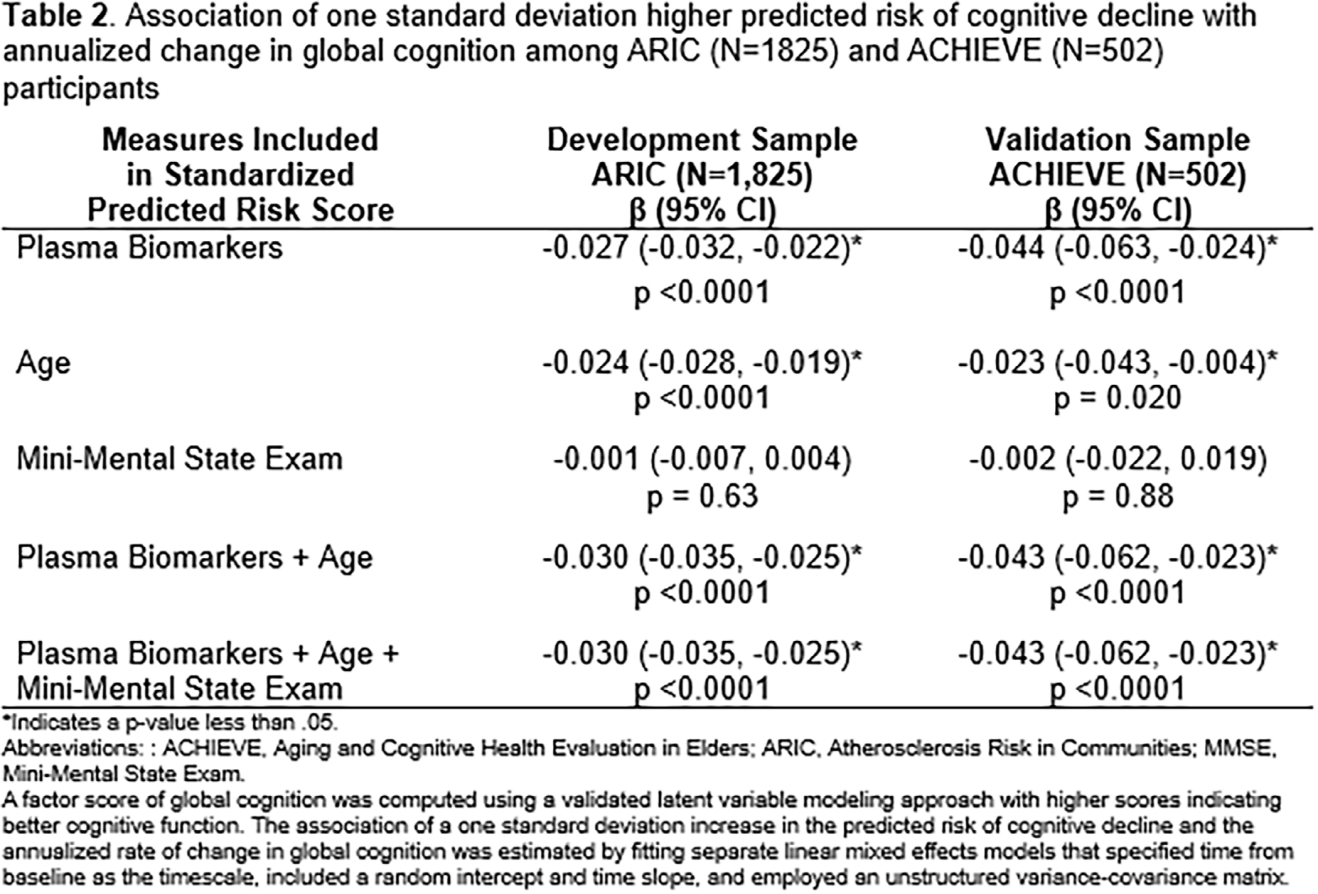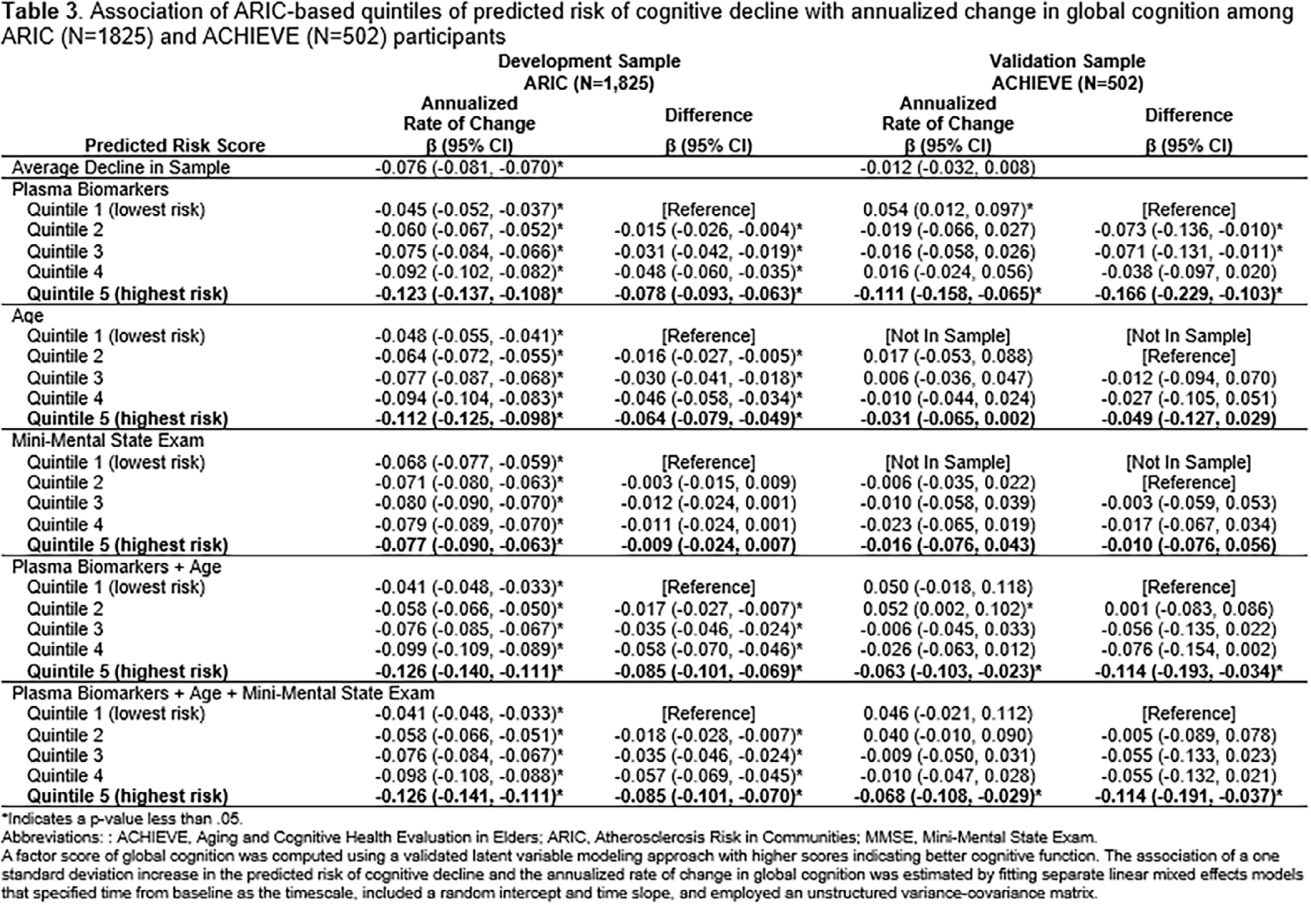# Identifying populations with a rapid rate of cognitive decline using plasma biomarkers

**DOI:** 10.1002/alz70856_105785

**Published:** 2026-01-08

**Authors:** Josef Coresh, James Russell Pike, Nicholas S Reed, Priya Palta, Jennifer A. Deal, Keenan A. Walker, Kevin J. Sullivan, Bharat Thyagarajan, Rebecca F. Gottesman, Thomas H. Mosley, Frank R Lin

**Affiliations:** ^1^ Departments of Population Health and Medicine, New York University Grossman School of Medicine, New York, NY, USA; ^2^ New York University, New York, NY, USA; ^3^ University of North Carolina at Chapel Hill, Chapel Hill, NC, USA; ^4^ Johns Hopkins Bloomberg School of Public Health, Baltimore, MD, USA; ^5^ Laboratory of Behavioral Neuroscience, National Institute on Aging, Intramural Research Program, Baltimore, MD, USA; ^6^ University of Mississippi Medical Center, The MIND Center, Jackson, MS, USA; ^7^ University of Minnesota, Minneapolis, MN, USA; ^8^ National Institute of Neurological Disorders & Stroke, Bethesda, MD, USA

## Abstract

**Background:**

Identifying populations with a rapid rate of cognitive decline is important for conducting effective clinical trials to slow down this rate of decline. This is important since healthy volunteers often have a shallower rate of cognitive decline and hence have little room for benefit resulting in underpowered clinical trials.

**Methods:**

In ARIC, we assayed frozen plasma collected in 2011‐13 using the Quanterix SiMoA platform. Neurodegeneration and AD biomarkers measured included amyloid‐β 42 to amyloid‐β 40 ratio, *p*‐tau 181, NFL, and GFAP. Global cognition factor score from ten neuropsychological tests were administered to ARIC participants from 2011‐2022. Linear mixed effects models with multiple imputation of pre‐death cognitive change estimated the association between biomarkers with annualized change in cognition. In ACHIEVE, assessed the 4 biomarkers using the Alamar CNS protein panel frozen plasma at the 3‐year follow‐up. Biomarkers were standardized within platforms using inverse‐normal transformation. Cognitive decline risk scores developed in ARIC were applied to ACHIEVE and tested as predictors of cognitive decline between years 3 and 5 of ACHIEVE.

**Results:**

Table 1 shows baseline characteristics and the annualized change in cognition, mean (SD) of ‐0.069 (0.087) among 1825 ARIC general population participants and much shallower and more variable ‐0.012 (0.231) among 502 ACHIEVE clinical trial volunteers. Biomarker risk scores developed in ARIC validated in ACHIEVE (Table 2) with the upper quintile (Table 3) having a mean (95% CI) progression rate of ‐0.123 (‐0.137, ‐0.108) in ARIC and ‐0.111 (‐0.158, ‐0.065) in ACHIEVE. Adding age and the Mini Mental State Exam did not improve the risk scores. The power of a clinical trial would change from 5% to 64% to 96% as mean annualized cognitive decline increases from ‐0.012 (volunteers selected like ACHIEVE) to ‐0.068 (general population like ARIC) to ‐0.111 (selected top risk quintile) assuming 800 participants, a progression SD of 0.25, and a 20% reduction in cognitive decline from an intervention.

**Conclusions:**

Risk scores based on plasma biomarkers may allow for the identification of individuals likely to experience rapid cognitive decline. Using biomarker‐based risk scores may increase the feasibility of future cognitive decline clinical trials.